# Understudied Anophelines Contribute to Malaria Transmission in a Low-Transmission Setting in the Choma District, Southern Province, Zambia

**DOI:** 10.4269/ajtmh.21-0989

**Published:** 2022-03-28

**Authors:** Mary E. Gebhardt, Kelly M. Searle, Tamaki Kobayashi, Timothy M. Shields, Harry Hamapumbu, Limonty Simubali, Twig Mudenda, Philip E. Thuma, Jennifer C. Stevenson, William J. Moss, Douglas E. Norris, for the Southern and Central Africa International Center of Excellence for Malaria Research

**Affiliations:** ^1^ Johns Hopkins Malaria Research Institute, The W. Harry Feinstone Department of Molecular Microbiology and Immunology, Johns Hopkins Bloomberg School of Public Health, Baltimore, Maryland;; ^2^ Division of Epidemiology and Community Health, University of Minnesota, Minneapolis, Minnesota;; ^3^ Department of Epidemiology, Johns Hopkins Bloomberg School of Public Health, Baltimore, Maryland;; ^4^ Macha Research Trust, Choma, Zambia

## Abstract

Malaria transmission has declined substantially in Southern Province, Zambia, which is considered a low-transmission setting. The Zambian government introduced a reactive test-and-treat strategy to identify active zones of transmission and treat parasitemic residents. This study was conducted in the Choma District, Southern Province, Zambia, concurrently with an evaluation of this strategy to identify vectors responsible for sustaining transmission, and to identify entomological, spatial, and ecological risk factors associated with increased densities of mosquitoes. Anophelines were collected with CDC light traps indoors and near animal pens in index cases and neighboring households. Outdoor collections captured significantly more anophelines than indoor traps, and 10 different anopheline species were identified. Four species (*Anopheles arabiensis*,* An. rufipes*,* An. squamosus*, and* An. coustani*) were positive for *Plasmodium falciparum* circumsporozoite protein by ELISA, and 61% of these 26 anophelines were captured outdoors. Blood meal assays confirm plasticity in *An. arabiensis* foraging, feeding both on humans and animals, whereas *An. rufipes*,* An. squamosus*, and *An. coustani* were largely zoophilic and exophilic. Linear regression of count data for indoor traps revealed that households with at least one parasitemic resident by polymerase chain reaction testing was associated with higher female anopheline counts. This suggests that targeting households with parasitemic individuals for vector interventions may reduce indoor anopheline populations. However, many vectors species responsible for transmission may not be affected by indoor interventions because they are primarily exophilic and forage opportunistically. These data underscore the necessity for further evaluation of vector surveillance and control tools that are effective outdoors, in conjunction with current indoor-based interventions.

## INTRODUCTION

Malaria remains one of the world’s greatest public health burdens. After decades of control efforts and a 50% worldwide reduction in malaria cases from 2005 to 2015, progress has stalled and malaria incidence has remained relatively stagnant, with an estimated 229 million cases worldwide in 2019.^1^^–^^3^ Progress has been heterogeneous across the African continent, with some regions still experiencing very high transmission and others that have reduced transmission dramatically.^1^ As some regions approach elimination, an increasing number of countries report continued malaria transmission and seasonal peaks in clinical cases even after significant coverage with the two most common indoor vector control tools: insecticide-treated nets (ITNs) and indoor residual spraying (IRS), as well as health clinics stocked with rapid diagnostic tests (RDTs) and artemisinin-based combination therapies.^4^^,^^5^ The success of ITNs and IRS rely on the long-held understanding of the biology of anopheline vectors to bite indoors and at night. It is unclear whether vectors of malaria in low-transmission settings transmit predominantly indoors and at night in settings with insufficient coverage, or if they use alternative foraging strategies that allow them to avoid these two indoor-based vector control tools.^4^^,^^6^^,^^7^

In the Choma District, Southern Province, Zambia, *Plasmodium falciparum* parasite prevalence decreased from 9% in 2008 to 1% in 2013 as measured by RDT, but low levels of malaria transmission still occur, with annual parasite prevalence ranging from 1% to 3% under active case surveillance.^8^^–^^10^ Since 2013, the government of Zambia has used a reactive test-and-treat strategy to help identify active zones of transmission and to treat parasitemic residents.^11^ According to this protocol, index cases are identified after testing positive for malaria by RDT at a local health facility. A health-care worker then follows up index cases with a visit to their household and every household within 140 m, testing every individual with an RDT, and providing treatment for anyone testing positive.^11^^,^^12^

The Southern and Central Africa International Center of Excellence for Malaria Research (ICEMR) has been working in this region for more than a decade and recognized the necessity to evaluate the effectiveness of this intervention strategy.^12^^,^^13^ From January 2015 to July 2018, the study team increased the screening radius of neighboring household visitation from 140 m to 250 m and used molecular diagnostics in addition to RDTs to evaluate whether this reactive test-and-treat method would capture effectively all local malaria transmission resulting from an index case.^12^^,^^13^ Results from parasite genomic studies indicated that this method did not reduce transmission to zero and did not address the composition of the vector population in this area. Although *Anopheles arabiensis* is generally considered to be the major vector because of its endophagic, anthropophilic nature, studies suggest that other species also may be playing an important role in sustaining low-level transmission.^14^^,^^15^

During this reactive test-and-treat evaluation, mosquitoes were collected both indoors and outdoors in animal pens in index cases and neighboring households to identify the vectors involved in malaria transmission. Further analyses of these data were performed with the intention to identify entomological, spatial, and ecological risk factors associated with increased densities of anopheline mosquitoes, both indoors and outdoors, in this low-transmission setting.

## MATERIALS AND METHODS

### Study region.

**Figure 1.**
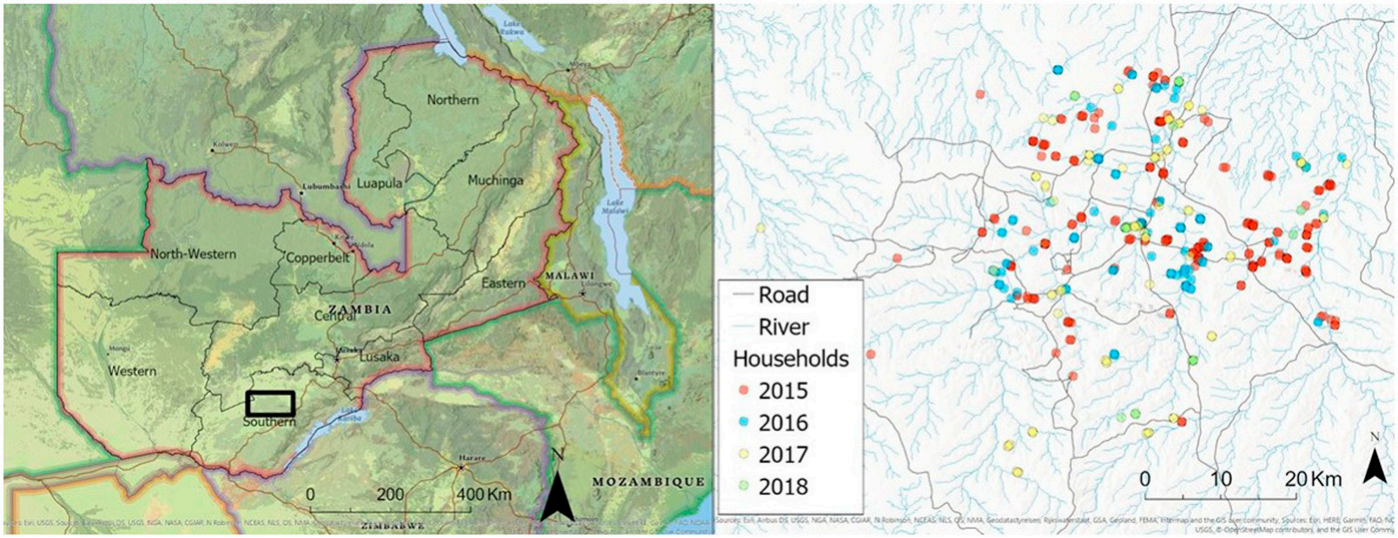
Study area and households sampled during study period (2015–2018). This figure appears in color at www.ajtmh.org.

The data for this study were collected from January 2015 to June 2018 in the catchment area of Macha Hospital, Choma District, Southern Province, Zambia (Figure 1). The region generally experiences three seasons: a rainy season from December to April, a cold dry season from May to August, and a hot dry season from September to November.^16^ *Anopheles arabiensis* is considered the major vector in the region, with numbers peaking during the rainy season.^14^ However, more recently our group has reported other *P. falciparum* circumsporozoite protein (CSP)-ELISA positive anopheline species in the area, including *An. squamosus*.^15^^,^^17^

### Reactive test-and-treat strategy.

The reactive test-and-treat program was initiated in the study area in 2013, and these methods were published previously.^11^^,^^12^ Briefly, when an individual tested positive for *P. falciparum* by RDT at a health-care facility, a local health-care worker traveled to the individual’s home and tested every person in the household—and every household within 140 m—with an RDT. For this study, the ICEMR team increased the radius of testing from 140 m to 250 m, and molecular diagnostics were used in addition to RDTs. If individuals were positive by RDT, they were treated with weight-dosed artemether/lumefantrine. Individual-level surveys were completed and parasite genetic studies have been reported.^10^

### Environmental covariates.

Global positioning satellite coordinates of households were recorded using a handheld global positioning satellite and were analyzed in ArcGIS Pro Version 2.4.0 (ESRI, Redlands, CA). Streams and rivers in the study area were categorized previously in order from 6 (the largest) to 1 (the smallest),^16^ and the distance from every trap to the closest stream category was measured using the ‘near’ tool in ArcGIS. Streams from orders 1 and 2 (henceforth 1/2) were combined for analysis, as were stream orders 3 and 4 (3/4), and 5 and 6 (5/6).

### Entomological sampling.

Mosquitoes were collected using a miniature CDC Light Trap (John W. Hock, Ltd., Gainesville, FL) in the index case household and at least one neighboring household from 1800 hr to 0600 hr. Selection of neighboring households was influenced by the proximity of the household to the index household, presence of an animal pen, and willingness to participate in the study. Traps were hung between 1.5 and 1.8 m off the ground, both next to a person sleeping under an ITN belonging to that household and outdoors next to an animal pen if one was present and traps were available. Household-level surveys were completed when traps were collected to gather information about entomological risk factors, including roof materials, open eaves, whether a fire was burned the night before, the number of ITNs, the number of people sleeping under ITNs, and the number of people sleeping in the house.

### Mosquito processing.

Mosquito samples were returned to Macha Research Trust, where they were identified morphologically according to Gillies and Coetzee,^18^ and stored individually on silica gel to desiccate. Each specimen was split into head/thorax and abdomen before proceeding with molecular processing to confirm species. DNA from female anopheline mosquito abdomens was extracted using a modified salt extraction as described previously.^19^ All specimens identified as *An. gambiae* or *An. funestus* underwent species-complex polymerase chain reaction (PCR) tests to determine species.^20^^–^^23^ All other specimens underwent a PCR test targeting the ribosomal DNA intergenic spacer region 2 (ITS2) as described previously for molecular confirmation of species.^23^ *Anopheles rufipes* and *An. pretoriensis* both result in a 500-bp band size in the ITS2 assay, so any specimen resulting in a 500-bp PCR product but not identified morphologically as *An. rufipes* or *An. pretoriensis* was considered *An. rufipes/pretoriensis*. For all analyses, *An. rufipes*,* An. pretoriensis*, and *An. rufipes/pretoriensis* were analyzed as a single group because of biological and behavioral similarities.^24^ As reported previously, *An. squamosus* does not produce a product in the ITS2 assay, so a modified molecular assay was used for molecular confirmation of *An. squamosus*.^25^

DNA from specimen abdomens that were CSP-ELISA positive were transported to the Johns Hopkins Bloomberg School of Public Health. The cytochrome oxidase I (COI) and ITS2 region of DNA were amplified, and specimens that produced a band were sent to the Johns Hopkins Medical Institutions Synthesis and Sequencing Facility for Sanger sequencing.^26^ Forward and reverse sequences were imported into Geneious Prime (version 2021.2.2, Biomatters, Ltd., Auckland, New Zealand, https://www.geneious.com), trimmed to remove low-quality reads, and aligned to create a consensus sequence. Consensus sequences were compared with the National Center for Biotechnology Information database using BLASTn, and final identifications were confirmed if they had more than 99% identity to a National Center for Biotechnology Information sequence. Sequences were submitted to GenBank, and accession numbers were provided for both ITS2 (OK050572-OK050581) and COI (OK017052-OK017066) (Supplemental Table S1).

### Host detection analyses.

To elucidate host preference, two different PCR assays were performed on female mosquito abdomens. For mosquitoes collected from 2015 to 2016, a mitochondrial cytochrome b PCR was used to detect host DNA.^19^ For mosquitoes collected from 2017 to 2018, a novel set of PCR primers targeting a “universal” vertebrate fragment of the 12S ribosomal RNA gene was used.^25^ An additional human-specific primer (Hum1F: 5’-CAC CAC GAT CAA AAG GGA CA-3’) was added to this 12S PCR, so samples with human DNA would produce two amplicons—the “universal” vertebrate product (205 bp) and a human product (541 bp) bands—instead of one. All samples of female anophelines from 2015 and 2016 that had an intact abdomen were processed to detect host DNA, whereas a subset of 2017 samples were analyzed because of the large number of samples. Samples were selected to reflect collection time and mosquito species abundance proportionately. All visibly blooded anophelines, 50% of indoor-caught females scored as not blooded and 10% of outdoor not-blooded females were selected for the subsample.

### Detection of sporozoites.

CSP-ELISAs were performed to detect the presence of *P. falciparum* sporozoites in head/thoraces at Macha Research Trust as described by the Malaria Research and Reference Reagent Resource Center. Samples were run in pools of five mosquito homogenates for the first ELISA, and then run individually if the pool was positive. Specimens were considered ELISA positive if the absorbance of the individual well was two times the absorbance of a negative insectary control mosquito.

### Risk factor analysis.

Two separate analyses were performed to identify covariates associated with the number of female anophelines captured per trap. The first analysis included only indoor traps that had matching epidemiological surveys (*n* = 370). Six households were excluded because they were missing data on coordinates or other variables of interest. The second analysis only included outdoor traps with matching epidemiological data (*n* = 145). One household was removed as a result of missing coordinates. Both analyses were performed using linear regressions of log-transformed mosquito count data in R.^27^ Multivariate analyses were performed using backward-selection step Akaike information criterion.

## RESULTS

### Household-level characteristics.

Between January 2015 and March 2018, a total of 402 unique households were visited, and mosquitoes were collected from 392 indoor traps, 88 cattle pen traps, and 67 goat pen traps. Index case households were represented in 44.4% of traps; neighboring households made up 49.7% of traps (5.9% of households with traps were of unknown status). More households were sampled during the rainy season each year, which is associated with a greater number of malaria cases (Supplemental Figure S1). A comprehensive table of the distribution of household characteristics for each trap placement can be viewed in Supplemental Table S2.

### Mosquito species composition.

A total of 5,282 female anopheline specimens were collected from a total of 547 trap-nights. The median and maximum number of female anophelines collected in outdoor traps (median, 2; interquartile range, 7; range, 0–1,661) was greater than indoor traps (median, 0; interquartile range, 2; range, 0–32) (*P* < 0.001, Supplemental Figure S2).

Ten different anopheline species were identified and, although all 10 species except *An. gambiae* s.s. were found both indoors and outdoors, the proportion of each species varied by trap placement (Table 1). Among indoor traps, *An. arabiensis* dominated catches, making up 70.0% of all female anophelines, whereas *An. squamosus* and *An. rufipes*/*An. pretoriensis* made up 79.7% of outdoor catches (Table 1). Other species collected included *An. coustani* (*n* = 192, 3.6%), *An. gambiae* s.s. (*n* = 2, 0.03%), *An. leesoni* (*n* = 20, 0.3%), *An. quadriannulatus* (*n* = 136, 2.6%), and *An. rivulorum*-like (*n* = 5, 0.09%).

**Table 1:** Anopheline species distribution by trap placement

Species	Indoor (*n* = 392)	Cattle pen (*n *= 88)	Goat pen (*n* = 67)	Total
*Anopheles arabiensis*	586	55	271	912
*An. coustani*	23	110	59	192
*An. gambiae *s.s.	2	0	0	2
*An. leesoni*	8	2	10	20
*An. longipalpis*	9	42	26	77
*An. pretoriensis*	10	176	151	337
*An. quadriannulatus*	24	22	90	136
*An. rivulorum*-like	1	0	4	5
*An. rufipes*	21	672	378	1,071
*An. rufipes*/*An. pretoriensis*	12	730	145	887
*An. squamosus*	83	974	317	1,374
Unidentified	57	118	94	269
Total	836	2,901*	1,545	5,282

* A total of 1,661 of 2,901 samples were collected from a single trap.

### Host preference.

Blood meal analysis using a cytochrome b-targeted PCR^19^ assay was performed on 1,598 of 1,622 samples (98.5%) from 2015 and 2016. An additional 459 of 3,485 samples (13.2%) from 2017 were analyzed for blood meal using a PCR protocol targeting 12S, which cannot differentiate animal species or mixed blood meals.^25^ Among all anophelines captured, 323 of 5,282 samples (6.1%) were recorded as visually blooded, and a host detection assay was performed on 286 visually blooded specimens, 1,696 visually not-blooded specimens, and 75 with unspecified blood status (Supplemental Table S3). Among the 2015 and 2016 samples that were visually blooded, host DNA was detected in 59%, whereas host DNA was detected in 86% of visually blooded anophelines from 2017. In addition, host DNA was detected in 26% of anophelines that were visually not blooded from 2015 and 2016, and in 15% of visually not blooded anophelines from 2017.

None of the species captured were exclusively human-biting, and animal DNA was detected more frequently than human DNA in all species except *An. arabiensis*, which was the predominant human forager of the mosquitoes sampled (Table 2). Of the 120 samples in which human DNA (either alone or in a mixed meal) was detected, 91 (75.8%) were *An. arabiensis* caught indoors. Other specimens that tested positive for human DNA were *An. arabiensis* caught outdoors (*n* = 3), *An. coustani* (*n* = 3), *An. quadriannulatus* (*n* = 2), *An. squamosus* (*n* = 7), *An. rufipes/pretoriensis* (*n* = 9), and *An. rivulorum*-like (*n* = 1). Among the 150 *An. arabiensis* specimen*s* in which host DNA was detected, 16% were found to have a mixed human–animal host DNA.

**Table 2:** Host DNA detection among various anopheline species

Species	Human	Mixed human and animal	Non-human animal*	Cow	Cow and goat	Goat	Pig	No fragment	Total
*Anopheles *arabiensis	70	24	9	21	0	26	0	405	555
*An. coustani*	3	0	6	1	0	3	0	36	49
*An. longipalpis*	0	0	0	0	0	4	0	31	35
*An. quadriannulatus*	2	0	1	5	0	6	0	57	71
*An. squamosus*	4	3	65	70	50	104	2	488	786
*An. rufipes *or* An. pretoriensis*	6	3	88	21	1	15	0	250	384
Other	1	0	0	1	0	0	0	23	25
Unidentified	3	1	1	7	3	12	0	125	152
Total	89	31	170	126	54	170	2	1,415	2,057

* Note that the assay used on the 2017 and 2018 samples could not identify mixed blood meals or vertebrate species contributing to the blood meal.

### ELISA results.

Of the 5,255 specimens run on CSP-ELISA, 26 (0.5%) were *P. falciparum* CSP positive from 10 different traps (Table 3). Anopheline species was confirmed by sequencing of ITS2 and COI fragments for 19 of 26 samples. At least four species were positive for CSP by ELISA: *An. arabiensis*,* An. coustani*,* An. rufipes*, and *An. squamosus* (Table 3). Ten of 26 (38.5%) of these specimens were collected from five indoor traps, 7 of 26 (26.9%) were collected from two traps placed near cattle pens, and 9 of 26 (34.6%) were collected from three traps placed near goat pens. All traps with at least one positive specimen came from different households.

**Table 3:** Circumsporozoite protein ELISA-positive individual anophelines

Species	*n*	Positive, %	Trap location	2015	2016	2017	Total
*Anopheles arabiensis*	911	1.54	Indoor	1	9	0	10
			Goat pen	0	4	0	4
*An. coustani*	192	0.52	Goat pen	0	0	1	1
*An. rufipes*	1,957*	0.10	Goat pen	0	2	0	2
*An. squamosus*	1,374	0.58	Goat pen	0	1	0	1
			Cattle pen	7	0	0	7
Unknown	–	–	Goat pen	0	1	0	1
Total	5,255	0.49	–	8	17	1	26

* This total includes all *An. rufipes* and *An. pretoriensis/rufipes*.

### Risk factor analysis: Indoor anopheline counts.

Among the 392 traps placed indoors, 363 (92.6%) also were accompanied by surveys completed at the household regarding household members and characteristics, and were included in the analysis. Univariate analyses indicated that covariates associated with increased mosquito counts indoors were the number of people sleeping in the house, the year 2016 and 2018, if someone in the house was PCR positive for *P. falciparum*, being an index case household, trapped during rainy season, and using a stream or pond as a water source (Table 4**)**. Having a head of household with a secondary or higher level of education was associated with a lower number of mosquitoes indoors.

**Table 4:** Results from univariate and multivariate logistic regression of logged anopheline count data from indoor collections

Variable	Univariate			Multivariate			
	Estimate	95% CI	*P* value	Estimate	95% CI	*P* value
People sleeping in house, *n*	0.023	–0.003 to 0.050	0.087 ns	0.036	0.010–0.062	0.007**
Distance to category 1/2 stream, km	–0.062	–0.241 to 0.159	0.550 ns	–	–	–
Distance to category 3/4 stream, km	0.026	–0.027 to 0.082	0.339 ns	–	–	–
Distance to category 5/6 stream, km	–0.011	–0.025 to 0.004	0.167 ns	–	–	–
Proportion sleeping under net	0.124	–0.088 to 0.387	0.272 ns	–	–	–
Median age, years	–0.005	–0.012 to 0.002	0.184 ns	–	–	–
Proportion female	–0.259	–0.506 to 0.113	0.148 ns	–0.328	[–0.543 to 0.013]	0.043*
Year							
2015	–	–	–	–	–	–	
2016	0.408	0.141–0.738	0.002**	0.600	0.203–1.130	0.001***
2017	–0.020	–0.224 to 0.237	0.864 ns	0.293	–0.056 to 0.771	0.109 ns
2018	0.606	0.038–1.486	0.033*	0.726	0.087–1.739	0.021*
Floor material							
Rudimentary	–	–	–	–	–	–
Finished	–0.199	–0.365 to 0.009	0.060 ns	–	–	–
At least one person PCR positive							
No	–	–	–	–	–	–
Yes	0.306	0.095–0.557	0.003**	0.196	0.010–0.416	0.038*
Index HH							
No	–	–	–	–	–	–
Yes	0.245	0.047–0.479	0.013*	–	–	–
Season							
Dry	–	–	–	–	–	–
Rainy	0.627	0.410–0.984	0.039*	0.777	0.477–1.139	< 0.001***
Water source							
Bore hole	–	–	–	–	–	–
Open well	0.237	–0.081 to 0.667	0.16 ns	0.303	–0.019 to 0.730	0.067 ns
Surface water	0.344	–0.017 to 0.839	0.064 ns	0.162	–0.138 to 0.567	0.323 ns
Stream/pond	0.332	0.065–0.667	0.012*	0.430	0.155–0.771	0.001***
Mixed/other	0.085	–0.250 to 0.571	0.662 ns	–0.057	–0.337 to 0.339	0.741 ns
Cooking tools							
Charcoal		–	–	–	–	–
Wood	0.151	–0.038 to 0.377	0.124 ns	–0.273	–0.449 to –0.041	0.024*
Mixed	–0.070	–0.446 to 0.559	0.781 ns	–0.264	–0.549 to 0.201	0.219 ns
Head of household education level							
Primary	–	–	–	–	–	–
Secondary	0.218	–0.025 to 0.520	0.082 ns	–	–	–
Higher	–0.430	– 0.655 to –0.058	0.028*	–	–	–
Eaves							
Closed	–	–	–	–	–	–
Open	0.000	–0.165 to 0.198	0.998 ns	–	–	–

HH = household; ns = not significant; PCR = polymerase chain reaction. * *P* value 0.01--0.05. ** *P* value 0.001--0.01. *** *P* value < 0.001.

In the multivariate analysis, having someone in the house who was *P. falciparum *PCR positive, year 2016 and 2017, trapped during the rainy season, the number of people in the house, and using a stream or pond as a water source remained associated positively with increased mosquito counts (Table 4). Interestingly, using wood for cooking compared with charcoal, and a greater proportion of women and girls in the home was associated with lower mosquito counts indoors.

### Risk factor analysis: Outdoor anopheline counts.

One hundred forty-five of 155 outdoor traps had epidemiological data and were included in the second analysis. In the univariate analysis, rainy season, the year 2017, increased distance from 5/6- and 3/4-order streams, and using wood for cooking were all associated positively with higher counts of anophelines in outdoor traps (Table 5). In the multivariate analysis, rainy season, the year 2017, distance from 3/4- and 5/6-order streams, and using a stream or pond as a water source were associated with increased anopheline counts (Table 5).

**Table 5:** Results from univariate and multivariate logistic regression of logged anopheline count data from outdoor collections

Variable	Univariate model			Multivariate model		
	Estimate	95% CI	*P* value	Estimate	95% CI	*P* value
Distance to category 1/2 stream, km	0.302	–0.342 to 1.576	0.445 ns	–	–	–
Distance to category 3/4 stream, km	0.181	0.018–0.370	0.028*	0.239	1.073–1.429	0.004**
Distance to category 5/6 stream, km	0.063	0.017–0.110	0.007**	0.060	1.011–1.110	0.016
People sleeping in house, *n*	0.020	–0.043 to 0.088	0.534 ns	–	–	–
Median age, years	0.006	–0.022 to 0.034	0.693 ns	–	–	–
Proportion female	–0.543	–0.886 to 0.825	0.265 ns			
Cooking tools						
Charcoal	–	–	–	–	–	–
Wood	0.852	0.052–2.262	0.033*	–	–	–
Mixed	–0.048	–0.714 to 2.175	0.936 ns	–	–	–
Water source						
Bore hole	–	–	–	–	–	–
Open well	–0.388	–0.773 to 0.646	0.328 ns	–0.236	0.302–1.935	0.568 ns
Surface water	0.824	–0.376 to 4.334	0.270 ns	0.033	0.382–2.787	0.950 ns
Stream/pond	0.142	–0.450 to 1.373	0.719 ns	1.635	1.257–5.529	0.011 ns
Mixed/other	–0.031	–0.616 to 1.446	0.947 ns	0.045	0.437 to 2.496	0.921 ns
Head of household education level						
Primary	–	–	–	–	–	–
Secondary	0.346	–0.344 to 1.762	0.415 ns	–	–	–
Higher	0.051	–0.957 to 24.772	0.976 ns	–	–	–
Season						
Dry	–	–	–	–	–	–
Rainy	1.790	0.652–3.709	< 0.001***	1.992	1.788–5.008	< 0.001***
Floor material						
Rudimentary	–	–	–	–	–	–
Finished	0.682	–0.142 to 2.298	0.129 ns	–	–	–
At least one person PCR positive						
No	–	–	–	–	–	–
Yes	–0.264	–0.576 to 0.276	–	–	–	–
Index HH						
No	–	–	–	–	–	–
Yes	–0.221	–0.543 to 0.325	–	–	–	–
Type of animal pen						
Goat pen	–	–	–	–	–	–
Cattle pen	–0.088	–0.469 to 0.566	–	–	–	–
Year						
2015	–	–	–	–	–	–
2016	0.023	–0.461 to 0.942	0.944 ns	0.259	0.657–2.410	0.486 ns
2017	1.818	0.468–4.415	0.002**	1.474	1.281–4.776	0.007**
2018	0.848	–0.461 to 5.329	0.326 ns	–0.151	0.230–3.131	0.804 ns

HH = household; ns = not significant; PCR = polymerase chain reaction. * *P* value 0.01--0.05. ** *P* value 0.001--0.01. *** *P* value < 0.001.

## DISCUSSION

In a region in southern Zambia with low malaria transmission, identifying vectors responsible for remaining transmission can be challenging. In our study, a reactive test-and-treat strategy was evaluated by collecting household information and mosquitos indoors and near animal pens. Ten species of anophelines were identified and verified molecularly, and the proportions of each species varied by trap placement. Four species were positive for *P. falciparum* sporozoites and were captured in both indoor and outdoor traps, adding to the growing body of evidence that risk for exposure to malaria vectors in southern Africa is not limited to indoor settings.^28^^–^^30^

Among indoor mosquitoes, the only species positive for sporozoites was *An. arabiensis*, which comprised up to 70% of indoor catches. Counts of this species are highly seasonal, peaking in the rainy season, which correlates with malaria transmission.^14^ However, in this region, *An. arabiensis* exhibit some opportunistic foraging behavior, and four CSP-positive *An. arabiensis* mosquitoes were captured outdoors in goat pens. Mixed human–animal host DNA was detected in 16% of *An. arabiensis*, further exemplifying this plasticity in host preference. In addition, 1.5% of captured *An. arabiensis* were positive for CSP, making it the vector with the greatest infection rate.

*Plasmodium falciparum *CSP was also detected in one *An. coustani*, two *An. rufipes*, and seven *An. squamosus*, all in outdoor animal pens. All three of these anopheline species are generally thought to be zoophilic and exophilic, but *An. rufipes* and *An. coustani* have been incriminated as vectors in other regions of Africa, associated with potential outdoor transmission and a range of human host preference.^30^^–^^32^ Host blood meal analysis for these species revealed a low human preference and a lower CSP positivity rate than *An. arabiensis.* However, their high relative counts make them a true threat to malaria elimination, especially in outdoor settings where ITNs and IRS are not applicable for vector control.

The rainy season and using a stream or pond as a water source were associated positively with greater mosquito densities in both indoor and outdoor traps. Considering the seasonality of malaria transmission, the increase in anophelines during the rainy season is expected and has been described in this area.^15^ Using streams or ponds as a water source might indicate that a household is closer to a breeding site or may be associated with lower socioeconomic status, which can be linked with greater malaria transmission resulting from factors such as lower quality housing and access to health care.^33^^,^^34^ However, when analyzing relationships using distance to stream order, no significant relationships were found for indoor anopheline densities, and a small relationship was found associating increased mosquito densities in outdoor traps with increased distance to 3/4- and 5/6-order streams.

In the indoor analysis, the number of people sleeping in a house, *P. falciparum *PCR-positive individuals in the household, index household, and the years 2016 and 2018 were also associated with increased indoor anopheline counts, whereas the proportion of female household members and higher education were associated with decreased indoor anopheline counts. Importantly, the association with households that had *P. falciparum *PCR-positive individuals indicates that targeting households with people who are parasitemic with vector control tools such as IRS and ITNs has the potential to affect the indoor vector populations.^35^ It is unclear in our analysis whether there is a causal relationship with the number of mosquitoes and households with at least one person positive for *P. falciparum*. It is likely that households with greater mosquito counts may cause more cases, rather than households with positive individuals attracting more mosquitoes.

One significant limitation of this study was that only households that were index or neighboring households were sampled for anophelines. This may have introduced geographic, temporal, and household structure bias in household selection. In addition, the selection of the neighboring households that were included was influenced by the availability of animal pens to set traps, which may introduce socioeconomic or *Anopheles* species bias. For future work, it is necessary to compare index and neighboring households to households in regions that do not have any reported malaria cases to determine whether these relationships persist.

In conclusion, we were able to provide evidence that despite indoor-based vector interventions, *An. arabiensis* is still involved in malaria transmission in Southern Province. However, understudied anophelines that are considered to be primarily exophagic, and therefore evade existing vector control interventions, appear to be playing a role as well and threaten the goal of malaria elimination.

## Supplemental Material


Supplemental materials
