# Supplementary material for: Understudied Anophelines Contribute to Malaria Transmission in a Low-Transmission Setting in the Choma District, Southern Province, Zambia
Source: Am J Trop Med Hyg. 2022 Mar 28;106(5):1406–13. doi: 10.4269/ajtmh.21-0989 (PMC9128685; doi:10.4269/ajtmh.21-0989)
Supplement: Supplementary file 1 [file tpmd210989.SD1.pdf]

Supplementary table 1. Molecular identification of ELISA positive mosquitoes

Collection Date	HH number	Sample ID	Trap location	Final ID	Individual ELISA	COI accession number	ITS2 Accession number
9-Feb-15	134	IcMa15_0141	indoor	arabiensis	positive	-	-
12-Feb-15	135	IcMa15_0142	cattle pen	squamosus	positive	OK017066	-
12-Feb-15	135	IcMa15_0143	cattle pen	squamosus	positive	OK017065	-
12-Feb-15	135	IcMa15_0145	cattle pen	squamosus	positive	OK017064	-
12-Feb-15	135	IcMa15_0173	cattle pen	squamosus	positive	OK017063	-
12-Feb-15	136	IcMa15_0554	cattle pen	squamosus	positive	-	-
12-Feb-15	136	IcMa15_0555	cattle pen	squamosus	positive	OK017062	-
12-Feb-15	136	IcMa15_0557	cattle pen	squamosus	positive	OK017061	-
20-Jan-16	479	IcMa16_0017	indoor	arabiensis	positive	OK017060	OK050581
11-Apr-16	588	IcMa16_0818	indoor	arabiensis	positive	OK017059	OK050580
11-Apr-16	588	IcMa16_0828	indoor	arabiensis	positive	OK017058	OK050579
11-Apr-16	588	IcMa16_0836	indoor	arabiensis	positive	OK017057	OK050578
11-Apr-16	588	IcMa16_0838	indoor	arabiensis	positive	OK017056	OK050577
11-Apr-16	634	IcMa16_0840	indoor	arabiensis	positive	OK017055	OK050576
20-Apr-16	645	IcMa16_0921	goat pen	rufipes	positive	OK017054	OK050575
20-Apr-16	645	IcMa16_0923	goat pen	rufipes	positive	OK017053	-
20-Apr-16	645	IcMa16_0925	goat pen	squamosus	positive	OK017052	-
20-Apr-16	645	IcMa16_0922	goat pen	unkonwn	positive	-	-
18-May-16	639	IcMa16_1018	indoor	arabiensis	positive	-	-
18-May-16	639	IcMa16_1019	indoor	arabiensis	positive	-	-
18-May-16	639	IcMa16_1020	indoor	arabiensis	positive	-	-
8-Jun-16	584	IcMa16_1062	goat pen	arabiensis	positive	-	OK050574
8-Jun-16	584	IcMa16_1063	goat pen	arabiensis	positive	-	-
8-Jun-16	584	IcMa16_1064	goat pen	arabiensis	positive	-	OK050573
8-Jun-16	584	IcMa16_1065	goat pen	arabiensis	positive	-	OK050572
22-Feb-17	779	IcMa17_0930	goat pen	coustani	positive	-	-

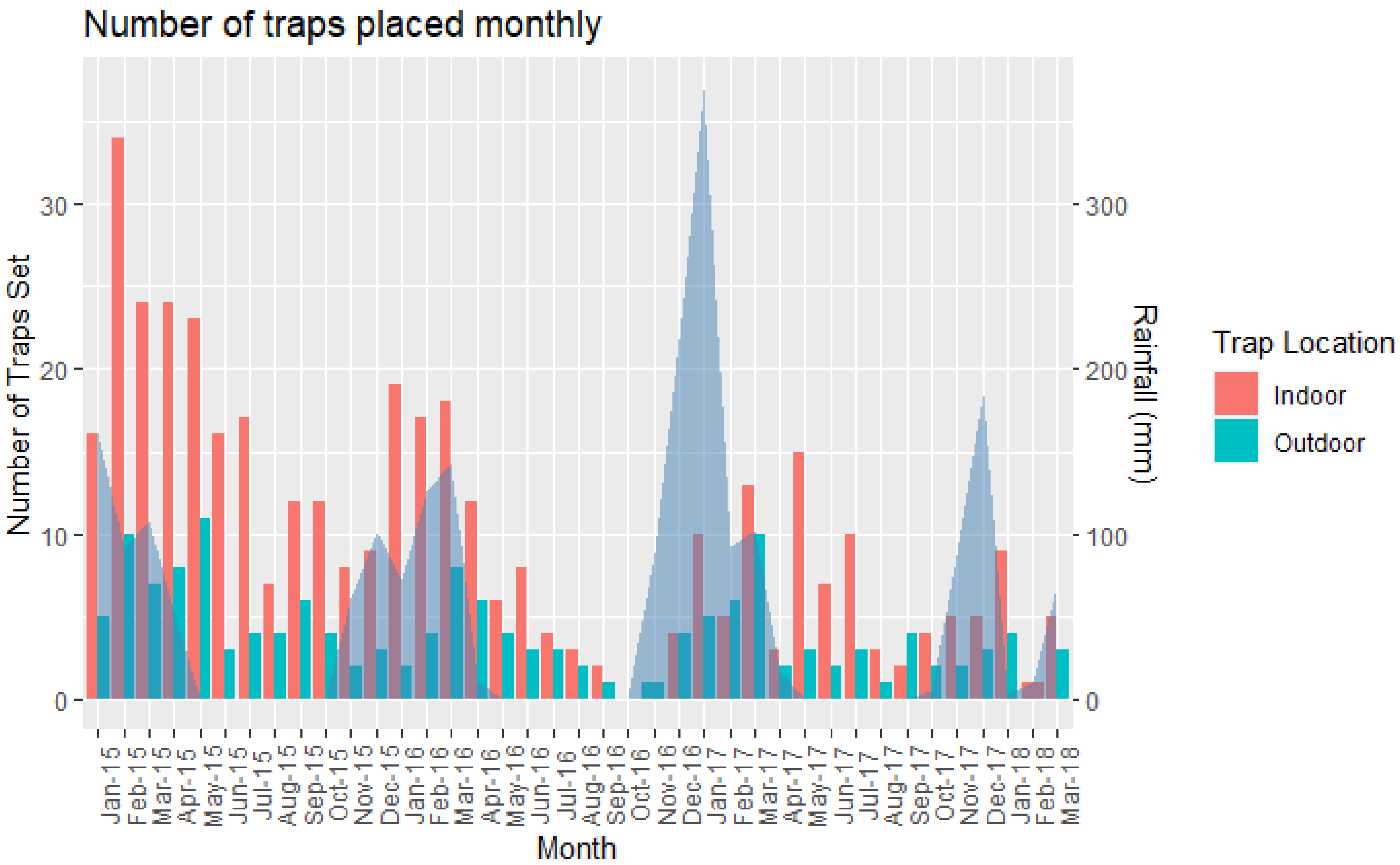

Supplementary figure 1. The number of indoor (orange) and outdoor (blue) traps placed each month. Blue shaded areas represent total monthly rainfall (mm)

Supplementary table 2. Household characteristics by trap placement.

Variable		Indoor	Cattle Pen	Goat Pen
		392	88	67
# people sleeping in HH (mean (SD))		5.63 (3.38)	-	-
Distance to category 1/2 stream (km) (mean (SD))		0.58 (0.41)	0.59 (0.40)	0.60 (0.41)
Distance to category 3/4 stream (km) (mean (SD))		2.23 (1.64)	2.45 (1.78)	2.12 (1.71)
Distance to category 5/6 stream (km) (mean (SD))		6.68 (5.98)	7.30 (6.24)	6.53 (5.88)
Proportion sleeping under net(mean (SD))		0.62 (0.42)	-	-
Median age in HH (mean (SD))		15.98 (12.10)	14.96 (9.73)	14.86 (9.58)
Prop. female (mean (SD))		0.53 (0.21)	0.50 (0.20)	0.50 (0.19)
# Female anophelines (mean (SD))		2.13 (5.01)	32.97 (179.96)	23.06 (64.94)
# Male anophelines (mean (SD))		0.02 (0.13)	0.09 (0.39)	0.07 (0.26)
# Total culicines (mean (SD))		4.35 (13.26)	28.57 (116.35)	44.40 (141.40)
Year (%)	2015	202 (51.5)	52 (59.1)	15 (22.4)
	2016	93 (23.7)	16 (18.2)	22 (32.8)
	2017	82 (20.9)	16 (18.2)	27 (40.3)
	2018	15 (3.8)	4 (4.5)	3 (4.5)
Season (%)	Rainy	241 (61.5)	54 (61.4)	41 (61.2)
	Dry	151 (38.5)	34 (38.6)	26 (38.8)
PCR positive individual in HH (%)	No	146 (37.2)	29 (33.0)	25 (37.3)
	Yes	223 (56.9)	56 (63.6)	35 (52.2)
	NA	23 (5.9)	3 (3.4)	7 (10.4)
Index HH (%)	No	195 (49.7)	42 (47.7)	33 (49.3)
	Yes	174 (44.4)	43 (48.9)	27 (40.3)
	NA	23 (5.9)	3 (3.4)	7 (10.4)
IRS sprayed HH(%)	No	70 (17.9)	0 (0.0)	0 (0.0)
	Yes	14 (3.6)	0 (0.0)	0 (0.0)
	NA	308 (78.6)	88 (100)	67 (100)
Roof type (%)	Metal	202 (51.5)	0 (0.0)	0 (0.0)
	Thatch	189 (48.2)	0 (0.0)	0 (0.0)
	NA	1 (0.3)	88 (100)	67 (100)
HH floor material (%)	Natural	284 (72.4)	63 (71.6)	41 (61.2)
	Finished	60 (15.3)	12 (13.6)	13 (19.4)
	NA	48 (12.2)	13 (14.8)	13 (19.4)
Eaves (%)	Closed	145 (37.0)	0 (0.0)	0 (0.0)
	Open	245 (62.5)	0 (0.0)	0 (0.0)
	NA	2 (0.5)	88 (100)	67 (100)
Water Source (%)	Bore hole/bush pump	202 (51.5)	49 (55.7)	35 (52.2)
	Open well	37 (9.4)	6 (6.8)	6 (9.0)
	Surface water	32 (8.2)	4 (4.5)	6 (9.0)
	Stream/pond	75 (19.1)	19 (21.6)	6 (9.0)
	mixed/other	22 (5.6)	7 (8.0)	7 (10.4)
	NA	24 (6.1)	3 (3.4)	7 (10.4)
Cooking materials (%)	Coal/charcoal	145 (37.0)	36 (40.9)	12 (17.9)
	Wood	212 (54.1)	43 (48.9)	46 (68.7)
	mixed	11 (2.8)	6 (6.8)	2 (3.0)
	NA	24 (6.1)	3 (3.4)	7 (10.4)
Highest education level in HH (%)	Primary	270 (68.9)	63 (71.6)	46 (68.7)
	Secondary	68 (17.3)	16 (18.2)	8 (11.9)
	Higher	11 (2.8)	0 (0.0)	1 (1.5)
	NA	43 (11.0)	9 (10.2)	12 (17.9)

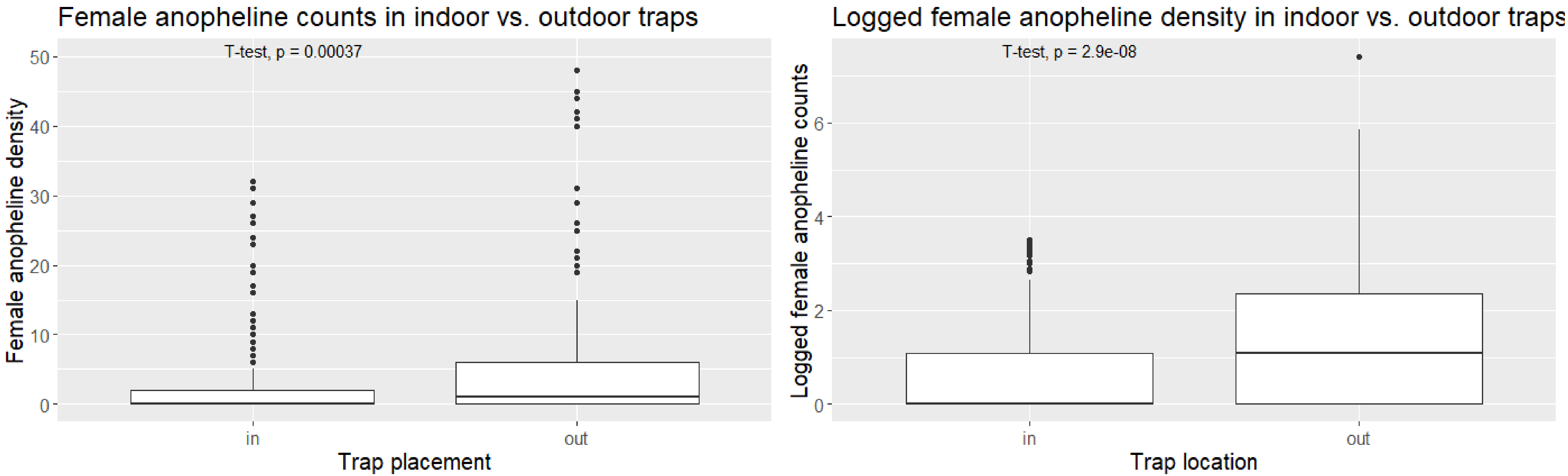

Supplementary figure 2. Counts and logged counts of female anophelines in indoor and outdoor traps. Note that figure a does not contain 14 outliers in the outdoor traps, ranging up to 1661 anophelines captured in a single cattle pen trap

Supplementary table 3. Host detection result by visually blooded status

Result	Visually blooded status		
	Blooded<br>n (%)	Not blooded<br>n (%)	Not specified<br>n (%)
Animal	126 (44.1)	43 (2.5)	1 (1.3)
Cow	32 (11.2)	92 (5.4)	2 (2.7)
Cow and Goat	14 (4.9)	39 (2.3)	1 (1.3)
Goat	12 (4.2)	155 (9.1)	3 (4.0)
Human	20 (7.0)	68 (4.0)	1 (1.3)
Pig	0 (0)	2 (0.1)	0 (0)
Mixed human and animal	8 (2.8)	0 (0)	1 (1.3)
No fragment	74 (25.9)	1275 (75.2)	66 (88)
**Total**	**286**	**1696**	**75**
